# The complete chloroplast genome sequence of *Berchemia berchemiifolia* (Rhamnaceae)

**DOI:** 10.1080/23802359.2018.1431068

**Published:** 2018-01-22

**Authors:** Kyeong-Sik Cheon, Kyung-Ah Kim, Ki-Oug Yoo

**Affiliations:** a Department of Biological Science, Sangji University, Wonju, Korea;; b Department of Biological Sciences, Kangwon National University, Chuncheon, Korea

**Keywords:** *Berchemia berchemiifolia*, chloroplast genome, rare plant, Rhamnaceae

## Abstract

The complete chloroplast genome sequence of *Berchemia berchemiifolia*, rare plant to Korea, was determined in this study. The total genome size was 160,410 bp in length, containing a pair of inverted repeats (IRs) of 26,514 bp, which were separated by a large single copy (LSC) and small single copy (SSC) of 88,627 bp and 18,755 bp, respectively. The overall GC contents of the chloroplast genome were 37.2%. One hundred twenty nine genes were annotated, including 84 protein coding genes, 37 tRNA genes, and 8 rRNA genes. In these genes, 18 genes contained one or two introns. The phylogenetic tree showed that *Berchemia berchemiifolia* was most closely related to *Berchemiella wilsonii*.

Genus *Berchemia*, belonging to Rhamnaceae, include about 32 taxa and distributed mainly in temperate and tropical regions of East to Southeast Asia (Michaux and Hedwig 2007). Among the *Berchemia* species, *Berchemia berchemiifolia* is distributed only in the Korea peninsula and Japan. In the Korea, especially, this species is distributed only in limited regions in Kyeongsangbuk-do and Chungcheongbuk-do Province, and three natural habitats in Chungcheongbuk-do Province are designated as natural monuments and protected. According to these reasons, it is listed in the rare plant in Korea as a ‘Vulnerable (VU)’ grade by IUCN category (Korea National Arboretum 2009).

In this study, we reported the complete chloroplast genome sequence of *B. berchemiifolia*, and this data will be used as an important information for protection strategy of species and the study of genome diversity. The plant materials were sampled from Sadam-ri (Goesan-gun, Chungcheongbuk-do Province, Korea). The voucher specimen has been deposited at the Kangwon National University Herbarium (KWNU93462). Total DNA was extracted using DNeasy Plant Mini Kit (Qiagen, Carlsbad, CA, USA). The genomic DNA was sequenced using the Miseq (Illumina Inc., San Diego, CA, USA). Using these sequence data, we assembled the complete chloroplast genome with Geneious 7.1.9 (Biomatters Ltd, New Zealand). Annotation was performed with Dual Organellar GenoMe Annotator (DOGMA) using default parameters to predict protein-coding genes, transfer RNA (tRNA) genes and ribosomal RNA (rRNA) genes (Wyman et al. 2004). BLASTX was used to further identify positions of genes with intron by searching against published plastid genome database. Genome map was drawn by OGDraw v 1.2 (Lohse et al. 2007). The complete cp genome sequence of *B. berchemiifolia* was submitted to GenBank under accession number MG739656.

The chloroplast genome of *B. berchemiifolia* was 160,410 bp in length with 37.2% GC content and composed of large single copy (LSC) region of 88,627 bp, small single copy (SSC) region of 18,755 bp, and two inverted repeat (IR) regions 26,514 bp. The gene content and order of plastid genome were almost identical to Rhamnaceae species (Ma et al. 2017; Wang et al. 2017) such as *Berchemiella wilsonii* (GenBank accession KY926621), *Ziziphus jujuba* (GenBank accession KU351660), and *Z. jujuba* var. *spinosa* (GenBank accession KX26630). A total of 129 genes were annotated, including 84 protein coding genes, 37 transfer RNA genes, and 8 ribosomal RNA genes. The *rps12* is trans-spliced gene with the 5′ exon located in the LSC region and 3′ exon was duplicated in the IR regions. Eighteen genes contain one or two introns (*trnA-UGC*, *trnG-UCC*, *trnI-GAU*, *trnK-UUU*, *trnL-UAA*, *trnV-UAC*, *petB*, *petD*, *atpF*, *ndhA*, *ndhB*, *clpP*, *rpl2*, *rpl16*, *rps12*, *rps16, rpoC1,* and *ycf3*).

To construct the phylogenetic tree, a total of 65 protein coding genes of 45 Rosales species and one outgroup (*Sophora alopecuroides*) were compiled into a single file of 74,041 bp and aligned with MAFFT (Katoh et al. 2002) and then, we conducted maximum likelihood (ML) analysis using RAxML v.7.4.2 with 1000 bootstrap replicates and GTR + I model (Stamatakis 2006). The phylogenetic tree (Figure 1) showed that Rhamnaceae are monophyletic and sister to Elaegnaceae. Also, *B. berchemiifolia* was most closely related to *Berchemiella wilsonii* with high support (BS = 100).

**Figure 1.**
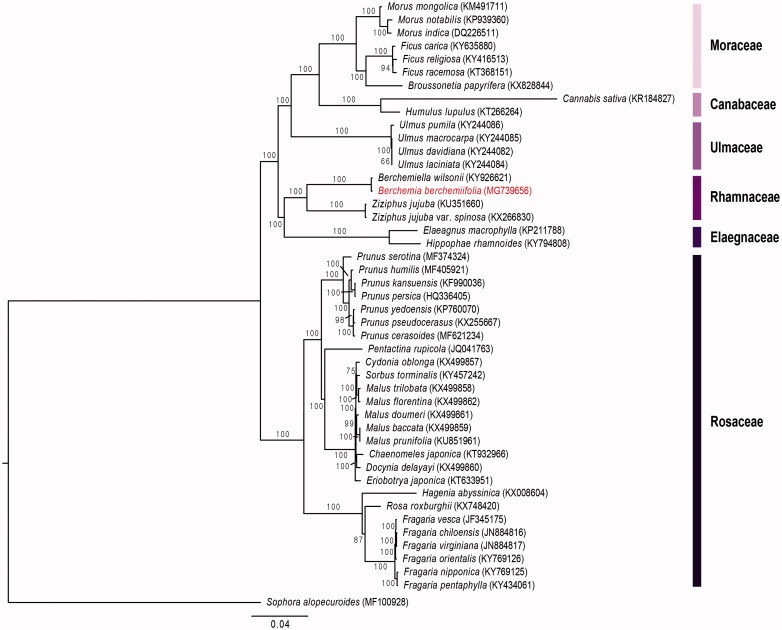
Maximum likelihood (ML) tree based on 65 protein-coding genes from 45 Rosales speceise and one outgroup (*Sophora alopecuroides*).
